# [2-(1,3-Benzothia­zol-2-ylmeth­oxy)-5-bromo­phen­yl](4-chloro­phen­yl)methanone

**DOI:** 10.1107/S1600536812049756

**Published:** 2012-12-12

**Authors:** Susanta K. Nayak, K. N. Venugopala, Thavendran Govender, Hendrik G. Kruger, Glenn E. M. Maguire

**Affiliations:** a Center for Nano Science and Technology@Polimi, Istituto Italiano di Tecnologia, Via Pascoli 70/3-20133 Milan, Italy; b School of Pharmacy and Pharmacology, University of Kwazulu-Natal, Durban 4000, South Africa; c School of Chemistry and Physics, University of KwaZulu-Natal, Durban 4000, South Africa

## Abstract

In the title compound, C_21_H_13_BrClNO_2_S, the dihedral angle between the planes of the benzothia­zole and chloro­phenyl­methanone groups is 71.34 (6)°. In the crystal, weak C—H⋯N hydrogen bonds lead to dimer formation, whereas Br⋯Cl short contacts [3.4966 (11) Å] form infinite chains along the *a*-axis direction. Further, the C—H⋯O, C—H⋯π and π–π [centroid–centroid distance = 3.865 (2) Å] inter­actions stabilize the three-dimensional network.

## Related literature

For background to the applications of benzothia­zole derivatives, see: Rana *et al.* (2007 ▶); Saeed *et al.* (2010 ▶); Telvekar *et al.* (2012 ▶); Venugopala *et al.* (2012 ▶). For their biological activity, see: Kelarev *et al.* (2003 ▶). For types of inter­actions involving halogens, see: Nayak *et al.* (2011 ▶).
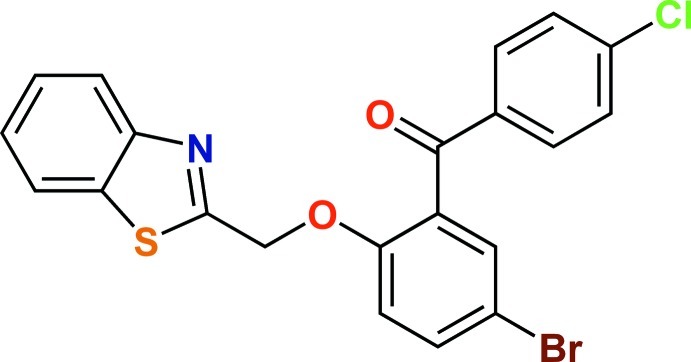


## Experimental

### 

#### Crystal data

C_21_H_13_BrClNO_2_S
*M* *_r_* = 458.74
Monoclinic, 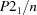

*a* = 13.7746 (3) Å
*b* = 7.4918 (2) Å
*c* = 18.7016 (7) Å
β = 106.013 (3)°
*V* = 1855.05 (10) Å^3^
*Z* = 4
Mo *K*α radiation
μ = 2.49 mm^−1^
*T* = 292 K
0.21 × 0.19 × 0.06 mm

#### Data collection

Oxford Diffraction Xcalibur (Eos, Nova) diffractometer
Absorption correction: multi-scan (*CrysAlis PRO*; Oxford Diffraction, 2009 ▶) *T*_min_ = 0.623, *T*_max_ = 0.865
19324 measured reflections
3645 independent reflections
2451 reflections with *I* > 2σ(*I*)
*R*_int_ = 0.054

#### Refinement

*R*[*F* ^2^ > 2σ(*F* ^2^)] = 0.044
*wR*(*F* ^2^) = 0.096
*S* = 1.07
3645 reflections
244 parameters
H-atom parameters constrained
Δρ_max_ = 0.45 e Å^−3^
Δρ_min_ = −0.43 e Å^−3^

### 

Data collection: *CrysAlis PRO* (Oxford Diffraction, 2009 ▶); cell refinement: *CrysAlis PRO*; data reduction: *CrysAlis PRO*; program(s) used to solve structure: *SHELXS97* (Sheldrick, 2008 ▶); program(s) used to refine structure: *SHELXL97* (Sheldrick, 2008 ▶); molecular graphics: *ORTEP-3 for Windows* (Farrugia, 2012) ▶ and *Mercury* (Macrae *et al.*, 2008 ▶); software used to prepare material for publication: *PLATON* (Spek, 2009 ▶) and *PARST* (Nardelli, 1995 ▶).

## Supplementary Material

Crystal structure: contains datablock(s) global, I. DOI: 10.1107/S1600536812049756/go2078sup1.cif

Structure factors: contains datablock(s) I. DOI: 10.1107/S1600536812049756/go2078Isup2.hkl

Supplementary material file. DOI: 10.1107/S1600536812049756/go2078Isup3.cml

Additional supplementary materials:  crystallographic information; 3D view; checkCIF report

## Figures and Tables

**Table 1:** Hydrogen-bond geometry (Å, °) *Cg*1 is the centroid of the thia­zole ring.

*D*—H⋯*A*	*D*—H	H⋯*A*	*D*⋯*A*	*D*—H⋯*A*
C5—H5⋯O2^i^	0.93	2.58	3.446 (4)	156
C17—H17⋯N1^ii^	0.93	2.61	3.434 (4)	147
C18—H18⋯*Cg*1^iii^	0.93	2.82	3.666 (3)	151

Symmetry codes: (i) 
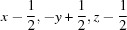
; (ii) 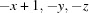; (iii) 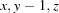.

**Fig. 1.**
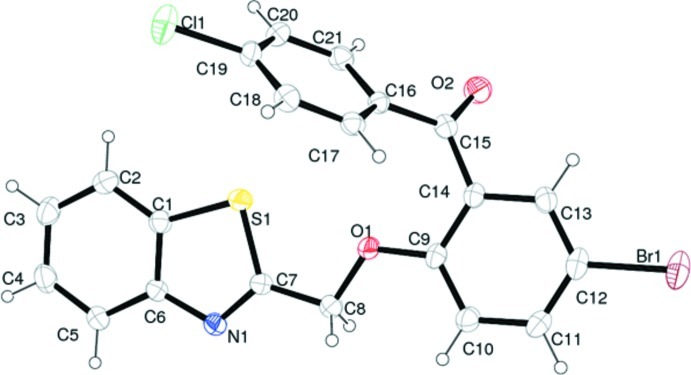
Molecular structure shows the atom labelling scheme with displacement ellipsoids for non-H atoms at 50% probability level, hydrogen atoms are arbitrary circle.

**Fig. 2.**
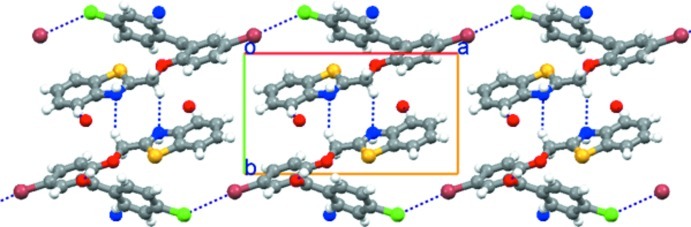
The C—H···N hydrogen bond dimers and Br···Cl short contacts of infinite chains along a axis.

**Fig. 3.**
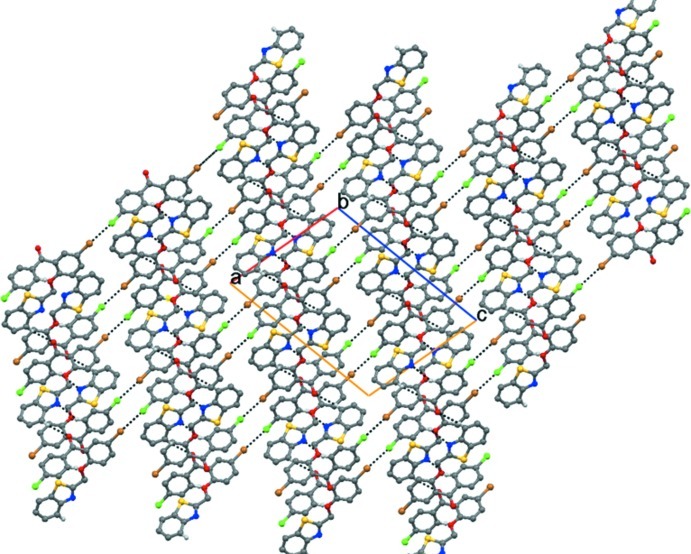
Sheets of three-dimensional network structure.

## supplementary crystallographic information

### Comment

Substituted benzothiazole derivatives have been reported to exhibit various pharmacological properties such as analgesic, antibacterial, antifungal, antidepressant, antitumor, antihypertensive, anthelmintic, and herbicidal activity (Kelarev *et al.*, 2003). However, the variety of biological features of new benzothiazole derivatives is of great scientific interest (Telvekar *et al.*, 2012; Saeed *et al.*, 2010). In continuation of our interest in synthesis and single-crystal analysis of benzothiazole molecule (Venugopala *et al.*, 2012), here we report the structure of the title compound.

The title compound prefers a conformation where the dihedral angle between the plane of the benzothiazole and the chlorophenyl methanone group is 71.34 (6)° (Fig. 2). The weak C17–H17···N1 hydrogen bonds (Table 1, Fig. 2) link the molecules to form a dimer. The C5–H5···O2, weak hydrogen bond, and the C18–H18···Cg1, C–H···π interaction, (Table 1), link the molecules into sheets which lie in the (101) plane and which run parallel to the *b*-axis, *Cg1* is the centroid of the five membered thiazole ring. This is stabilized by the π–π interaction, *Cg2*···*Cg3*, (-*x*+1, -*y*, -*z*), in which the centroid to centroid distance is 3.865 (2) Å, the dihedral angle between the planes is 9.49 (15)° and the perpendicular distance between *Cg2* on to the plane of the ring with centroid *Cg3* is 3.3415 (14)Å, *Cg2* is the centroid of the six membered ring containing atoms C1 to C6 and *Cg3* is the centroid of six membered ring containing atoms C9 to C14. A Br···Cl short contact links these sheets along the *a* axis to give a three-dimensional network (Fig. 3). The Br1···Cl1 distance = 3.4966 (11)Å, Br1···Cl1(*x* + 1, *y* , *z*) and Cl1···.Br1(*x* - 1, *y*, *z*); angle at Br1, C12–Br1···Cl1(*x* + 1, *y* , *z*) = 173.56 (13)°; angle at Cl1, C19–Cl1···Br1(*x* - 1, *y* , *z*) = 138.2 (2)°: Type II; Nayak *et al.*, 2011].

### Experimental

To a solution of (5-bromo-2-hydroxyphenyl)(4-chlorophenyl) methanone (1 mmol) and (2-chloromethyl)benzo[*d*]thiazole (1 mmol) in dry THF, dry potassium carbonate (1 mmol) was added and stirred at room temperature for 8 h. The reaction mixture was concentrated to remove the solvent, diluted with ethyl acetate, washed with water, brine solution and dried over anhydrous sodium sulfate. The organic layer was concentrated to yield a residue which was purified by column chromatography using ethyl acetate and n-hexane as eluent (7:3, Rf = 3/4) to afford the product in 64% as a brown solid (m. p. 450 K). Suitable crystals for single-crystal X-ray study were obtained from ethanol solvent using slow evaporation technique at room temperature.

### Refinement

All H atoms were positioned geometrically and refined using a riding model with *U*_iso_(H)= 1.2 *U*_eq_(C).

### Crystal data

**Table:**

C_21_H_13_BrClNO_2_S	*F*(000) = 920
*M**_r_* = 458.74	*D*_x_ = 1.643 Mg m^−^^3^
Monoclinic, *P*2_1_/*n*	Mo *K*α radiation, λ = 0.7107 Å
Hall symbol: -P 2yn	Cell parameters from 450 reflections
*a* = 13.7746 (3) Å	θ = 1.0–28.0°
*b* = 7.4918 (2) Å	µ = 2.49 mm^−^^1^
*c* = 18.7016 (7) Å	*T* = 292 K
β = 106.013 (3)°	Plate, colourless
*V* = 1855.05 (10) Å^3^	0.21 × 0.19 × 0.06 mm
*Z* = 4	

### Data collection

**Table:**

Oxford Diffraction Xcalibur (Eos, Nova) diffractometer	3645 independent reflections
Radiation source: Mova (Mo) X-ray Source	2451 reflections with *I* > 2σ(*I*)
Mirror monochromator	*R*_int_ = 0.054
Detector resolution: 16.0839 pixels mm^-1^	θ_max_ = 26.0°, θ_min_ = 3.0°
ω scans	*h* = −16→16
Absorption correction: multi-scan (*CrysAlis PRO*; Oxford Diffraction, 2009)	*k* = −9→9
*T*_min_ = 0.623, *T*_max_ = 0.865	*l* = −23→23
19324 measured reflections	

### Refinement

**Table:**

Refinement on *F*^2^	Primary atom site location: structure-invariant direct methods
Least-squares matrix: full	Secondary atom site location: difference Fourier map
*R*[*F*^2^ > 2σ(*F*^2^)] = 0.044	Hydrogen site location: inferred from neighbouring sites
*wR*(*F*^2^) = 0.096	H-atom parameters constrained
*S* = 1.07	*w* = 1/[σ^2^(*F*_o_^2^) + (0.0325*P*)^2^ + 0.1234*P*] where *P* = (*F*_o_^2^ + 2*F*_c_^2^)/3
3645 reflections	(Δ/σ)_max_ = 0.001
244 parameters	Δρ_max_ = 0.45 e Å^−^^3^
0 restraints	Δρ_min_ = −0.43 e Å^−^^3^

### Special details

**Table:**

Experimental. CrysAlisPro, Oxford Diffraction Ltd., Version 1.171.33.34d Empirical absorption correction using spherical harmonics, implemented in SCALE3 ABSPACK scaling algorithm.
Geometry. All s.u.'s (except the s.u. in the dihedral angle between two l.s. planes) are estimated using the full covariance matrix. The cell s.u.'s are taken into account individually in the estimation of s.u.'s in distances, angles and torsion angles; correlations between s.u.'s in cell parameters are only used when they are defined by crystal symmetry. An approximate (isotropic) treatment of cell s.u.'s is used for estimating s.u.'s involving l.s. planes.
Refinement. Refinement of *F*^2^ against ALL reflections. The weighted *R*-factor *wR* and goodness of fit *S* are based on *F*^2^, conventional *R*-factors *R* are based on *F*, with *F* set to zero for negative *F*^2^. The threshold expression of *F*^2^ > 2σ(*F*^2^) is used only for calculating *R*-factors(gt) *etc*. and is not relevant to the choice of reflections for refinement. *R*-factors based on *F*^2^ are statistically about twice as large as those based on *F*, and *R*- factors based on ALL data will be even larger.

### Fractional atomic coordinates and isotropic or equivalent isotropic displacement parameters (Å^2^)

**Table:**

	*x*	*y*	*z*	*U*_iso_*/*U*_eq_	
Br1	1.04455 (3)	−0.14743 (5)	0.15995 (3)	0.06540 (18)	
S1	0.41791 (7)	0.14738 (11)	0.12108 (5)	0.0461 (2)	
Cl1	0.28248 (7)	−0.33938 (14)	0.18242 (7)	0.0736 (3)	
O1	0.61465 (16)	0.0996 (3)	0.11015 (12)	0.0437 (6)	
N1	0.39940 (19)	0.3398 (3)	0.00334 (14)	0.0356 (6)	
O2	0.75803 (17)	−0.0576 (3)	0.30851 (13)	0.0563 (7)	
C14	0.7576 (2)	−0.0504 (4)	0.18309 (18)	0.0351 (8)	
C6	0.3056 (2)	0.3399 (4)	0.01745 (17)	0.0337 (7)	
C9	0.7118 (2)	0.0447 (4)	0.11796 (18)	0.0359 (8)	
C17	0.5611 (2)	−0.2566 (4)	0.16701 (16)	0.0363 (8)	
H17	0.5988	−0.2865	0.1345	0.044*	
C16	0.6032 (2)	−0.1492 (4)	0.22765 (17)	0.0320 (7)	
C1	0.3006 (2)	0.2404 (4)	0.07985 (17)	0.0393 (8)	
C20	0.4476 (2)	−0.1627 (4)	0.26215 (18)	0.0427 (8)	
H20	0.4088	−0.1295	0.2935	0.051*	
C7	0.4624 (2)	0.2449 (4)	0.05198 (16)	0.0341 (7)	
C10	0.7655 (2)	0.0818 (4)	0.06722 (19)	0.0432 (8)	
H10	0.7350	0.1464	0.0244	0.052*	
C8	0.5692 (2)	0.2169 (4)	0.05054 (18)	0.0416 (8)	
H8A	0.5711	0.1649	0.0035	0.050*	
H8B	0.6050	0.3298	0.0567	0.050*	
C15	0.7089 (2)	−0.0826 (4)	0.24430 (19)	0.0366 (8)	
C18	0.4637 (2)	−0.3200 (4)	0.15417 (19)	0.0414 (8)	
H18	0.4363	−0.3952	0.1141	0.050*	
C5	0.2192 (2)	0.4269 (4)	−0.02485 (18)	0.0432 (8)	
H5	0.2210	0.4945	−0.0662	0.052*	
C19	0.4081 (2)	−0.2704 (4)	0.20142 (19)	0.0409 (8)	
C11	0.8638 (2)	0.0239 (4)	0.0796 (2)	0.0447 (9)	
H11	0.8993	0.0471	0.0448	0.054*	
C2	0.2111 (3)	0.2240 (5)	0.09968 (19)	0.0507 (9)	
H2	0.2083	0.1569	0.1409	0.061*	
C21	0.5458 (2)	−0.1054 (4)	0.27540 (18)	0.0409 (8)	
H21	0.5743	−0.0360	0.3172	0.049*	
C4	0.1317 (3)	0.4104 (5)	−0.0041 (2)	0.0542 (10)	
H4	0.0739	0.4687	−0.0317	0.065*	
C13	0.8576 (2)	−0.1043 (4)	0.19534 (19)	0.0394 (8)	
H13	0.8898	−0.1650	0.2388	0.047*	
C12	0.9090 (2)	−0.0684 (4)	0.1436 (2)	0.0454 (9)	
C3	0.1270 (3)	0.3092 (5)	0.0570 (2)	0.0572 (10)	
H3	0.0661	0.2991	0.0691	0.069*	

### Atomic displacement parameters (Å^2^)

**Table:**

	*U*^11^	*U*^22^	*U*^33^	*U*^12^	*U*^13^	*U*^23^
Br1	0.0341 (2)	0.0652 (3)	0.0997 (4)	0.00450 (18)	0.0232 (2)	−0.0024 (2)
S1	0.0439 (5)	0.0521 (6)	0.0458 (6)	0.0111 (4)	0.0182 (5)	0.0180 (4)
Cl1	0.0384 (5)	0.0839 (8)	0.0968 (9)	−0.0133 (5)	0.0160 (6)	−0.0076 (6)
O1	0.0339 (12)	0.0514 (14)	0.0502 (15)	0.0117 (10)	0.0188 (12)	0.0197 (11)
N1	0.0365 (15)	0.0345 (15)	0.0351 (16)	0.0021 (12)	0.0087 (13)	0.0025 (12)
O2	0.0474 (15)	0.0796 (18)	0.0373 (15)	−0.0139 (13)	0.0040 (13)	−0.0010 (13)
C14	0.0302 (17)	0.0320 (18)	0.044 (2)	0.0009 (14)	0.0111 (16)	−0.0017 (16)
C6	0.0363 (18)	0.0317 (17)	0.0325 (18)	0.0029 (14)	0.0087 (15)	−0.0003 (15)
C9	0.0304 (17)	0.0326 (18)	0.047 (2)	0.0010 (14)	0.0139 (16)	−0.0020 (15)
C17	0.040 (2)	0.0357 (19)	0.0344 (19)	0.0064 (15)	0.0124 (16)	0.0029 (15)
C16	0.0329 (17)	0.0315 (17)	0.0323 (18)	0.0029 (14)	0.0100 (15)	0.0021 (15)
C1	0.0385 (19)	0.0397 (19)	0.040 (2)	0.0061 (15)	0.0115 (17)	0.0021 (16)
C20	0.039 (2)	0.051 (2)	0.042 (2)	0.0012 (16)	0.0183 (18)	0.0030 (17)
C7	0.0379 (18)	0.0327 (18)	0.0324 (18)	0.0016 (15)	0.0106 (16)	0.0007 (15)
C10	0.041 (2)	0.0414 (19)	0.050 (2)	0.0049 (16)	0.0178 (18)	0.0057 (16)
C8	0.0389 (19)	0.043 (2)	0.044 (2)	0.0073 (16)	0.0132 (17)	0.0108 (17)
C15	0.0364 (18)	0.0324 (17)	0.039 (2)	0.0002 (14)	0.0068 (17)	−0.0007 (15)
C18	0.043 (2)	0.037 (2)	0.042 (2)	−0.0027 (15)	0.0072 (18)	−0.0031 (16)
C5	0.0387 (19)	0.049 (2)	0.039 (2)	0.0038 (17)	0.0068 (17)	0.0043 (16)
C19	0.0290 (18)	0.043 (2)	0.049 (2)	−0.0034 (15)	0.0074 (17)	0.0082 (17)
C11	0.041 (2)	0.044 (2)	0.056 (2)	−0.0013 (16)	0.0252 (19)	−0.0015 (18)
C2	0.048 (2)	0.060 (2)	0.049 (2)	0.0020 (19)	0.021 (2)	0.0089 (19)
C21	0.044 (2)	0.045 (2)	0.0316 (19)	−0.0023 (16)	0.0077 (17)	−0.0041 (15)
C4	0.038 (2)	0.070 (3)	0.050 (2)	0.0113 (19)	0.0049 (19)	−0.003 (2)
C13	0.0322 (18)	0.0383 (19)	0.044 (2)	−0.0004 (14)	0.0048 (17)	0.0014 (15)
C12	0.0314 (18)	0.0378 (19)	0.068 (3)	−0.0006 (15)	0.0156 (19)	−0.0073 (19)
C3	0.038 (2)	0.075 (3)	0.063 (3)	0.004 (2)	0.020 (2)	−0.001 (2)

### Geometric parameters (Å, º)

**Table:**

Br1—C12	1.902 (3)	C20—C19	1.377 (4)
S1—C1	1.733 (3)	C20—H20	0.9300
S1—C7	1.737 (3)	C7—C8	1.493 (4)
Cl1—C19	1.746 (3)	C10—C11	1.380 (4)
O1—C9	1.368 (3)	C10—H10	0.9300
O1—C8	1.422 (3)	C8—H8A	0.9700
N1—C7	1.285 (4)	C8—H8B	0.9700
N1—C6	1.389 (4)	C18—C19	1.371 (4)
O2—C15	1.219 (3)	C18—H18	0.9300
C14—C13	1.392 (4)	C5—C4	1.370 (5)
C14—C9	1.402 (4)	C5—H5	0.9300
C14—C15	1.498 (4)	C11—C12	1.374 (4)
C6—C5	1.395 (4)	C11—H11	0.9300
C6—C1	1.402 (4)	C2—C3	1.370 (5)
C9—C10	1.383 (4)	C2—H2	0.9300
C17—C18	1.380 (4)	C21—H21	0.9300
C17—C16	1.381 (4)	C4—C3	1.388 (5)
C17—H17	0.9300	C4—H4	0.9300
C16—C21	1.385 (4)	C13—C12	1.373 (4)
C16—C15	1.489 (4)	C13—H13	0.9300
C1—C2	1.387 (4)	C3—H3	0.9300
C20—C21	1.375 (4)		
			
C1—S1—C7	88.70 (15)	H8A—C8—H8B	108.5
C9—O1—C8	118.4 (2)	O2—C15—C16	120.2 (3)
C7—N1—C6	110.4 (3)	O2—C15—C14	118.9 (3)
C13—C14—C9	118.6 (3)	C16—C15—C14	120.9 (3)
C13—C14—C15	117.3 (3)	C19—C18—C17	118.9 (3)
C9—C14—C15	123.8 (3)	C19—C18—H18	120.5
N1—C6—C5	125.8 (3)	C17—C18—H18	120.5
N1—C6—C1	114.8 (3)	C4—C5—C6	118.5 (3)
C5—C6—C1	119.4 (3)	C4—C5—H5	120.8
O1—C9—C10	124.0 (3)	C6—C5—H5	120.8
O1—C9—C14	116.0 (3)	C18—C19—C20	121.8 (3)
C10—C9—C14	120.0 (3)	C18—C19—Cl1	119.2 (3)
C18—C17—C16	120.7 (3)	C20—C19—Cl1	119.0 (3)
C18—C17—H17	119.6	C12—C11—C10	119.4 (3)
C16—C17—H17	119.6	C12—C11—H11	120.3
C17—C16—C21	118.9 (3)	C10—C11—H11	120.3
C17—C16—C15	122.1 (3)	C3—C2—C1	118.3 (3)
C21—C16—C15	119.0 (3)	C3—C2—H2	120.8
C2—C1—C6	121.4 (3)	C1—C2—H2	120.8
C2—C1—S1	129.3 (3)	C20—C21—C16	121.2 (3)
C6—C1—S1	109.3 (2)	C20—C21—H21	119.4
C21—C20—C19	118.5 (3)	C16—C21—H21	119.4
C21—C20—H20	120.8	C5—C4—C3	121.8 (3)
C19—C20—H20	120.8	C5—C4—H4	119.1
N1—C7—C8	122.8 (3)	C3—C4—H4	119.1
N1—C7—S1	116.8 (2)	C12—C13—C14	120.4 (3)
C8—C7—S1	120.4 (2)	C12—C13—H13	119.8
C11—C10—C9	120.6 (3)	C14—C13—H13	119.8
C11—C10—H10	119.7	C13—C12—C11	121.0 (3)
C9—C10—H10	119.7	C13—C12—Br1	120.0 (3)
O1—C8—C7	107.2 (2)	C11—C12—Br1	119.0 (3)
O1—C8—H8A	110.3	C2—C3—C4	120.6 (4)
C7—C8—H8A	110.3	C2—C3—H3	119.7
O1—C8—H8B	110.3	C4—C3—H3	119.7
C7—C8—H8B	110.3		
			
C7—N1—C6—C5	−179.0 (3)	C21—C16—C15—C14	−151.8 (3)
C7—N1—C6—C1	0.3 (4)	C13—C14—C15—O2	40.1 (4)
C8—O1—C9—C10	−6.7 (4)	C9—C14—C15—O2	−133.8 (3)
C8—O1—C9—C14	171.5 (3)	C13—C14—C15—C16	−138.1 (3)
C13—C14—C9—O1	−177.8 (3)	C9—C14—C15—C16	48.0 (4)
C15—C14—C9—O1	−4.0 (4)	C16—C17—C18—C19	1.9 (5)
C13—C14—C9—C10	0.5 (4)	N1—C6—C5—C4	178.6 (3)
C15—C14—C9—C10	174.3 (3)	C1—C6—C5—C4	−0.6 (5)
C18—C17—C16—C21	−0.2 (4)	C17—C18—C19—C20	−1.5 (5)
C18—C17—C16—C15	178.3 (3)	C17—C18—C19—Cl1	176.3 (2)
N1—C6—C1—C2	−178.2 (3)	C21—C20—C19—C18	−0.5 (5)
C5—C6—C1—C2	1.1 (5)	C21—C20—C19—Cl1	−178.3 (2)
N1—C6—C1—S1	0.5 (3)	C9—C10—C11—C12	−1.3 (5)
C5—C6—C1—S1	179.8 (2)	C6—C1—C2—C3	−0.5 (5)
C7—S1—C1—C2	177.7 (3)	S1—C1—C2—C3	−178.9 (3)
C7—S1—C1—C6	−0.8 (2)	C19—C20—C21—C16	2.2 (5)
C6—N1—C7—C8	178.9 (3)	C17—C16—C21—C20	−1.8 (5)
C6—N1—C7—S1	−1.0 (3)	C15—C16—C21—C20	179.6 (3)
C1—S1—C7—N1	1.1 (3)	C6—C5—C4—C3	−0.5 (5)
C1—S1—C7—C8	−178.8 (3)	C9—C14—C13—C12	−1.5 (5)
O1—C9—C10—C11	179.0 (3)	C15—C14—C13—C12	−175.7 (3)
C14—C9—C10—C11	0.9 (5)	C14—C13—C12—C11	1.2 (5)
C9—O1—C8—C7	176.2 (2)	C14—C13—C12—Br1	−178.6 (2)
N1—C7—C8—O1	−177.4 (3)	C10—C11—C12—C13	0.3 (5)
S1—C7—C8—O1	2.4 (4)	C10—C11—C12—Br1	180.0 (2)
C17—C16—C15—O2	−148.5 (3)	C1—C2—C3—C4	−0.5 (5)
C21—C16—C15—O2	30.0 (4)	C5—C4—C3—C2	1.1 (6)
C17—C16—C15—C14	29.7 (4)		

### Hydrogen-bond geometry (Å, º)

Cg1 is the centroid of the thiazole ring.

**Table:**

*D*—H···*A*	*D*—H	H···*A*	*D*···*A*	*D*—H···*A*
C5—H5···O2^i^	0.93	2.58	3.446 (4)	156
C17—H17···N1^ii^	0.93	2.61	3.434 (4)	147
C18—H18···*Cg*1^iii^	0.93	2.82	3.666 (3)	151

Symmetry codes: (i) *x*−1/2, −*y*+1/2, *z*−1/2; (ii) −*x*+1, −*y*, −*z*; (iii) *x*, *y*−1, *z*.
